# Integration of Colloidal Quantum Dots with Photonic Structures for Optoelectronic and Optical Devices

**DOI:** 10.1002/advs.202101560

**Published:** 2021-07-28

**Authors:** Mengyu Chen, Lihua Lu, Hui Yu, Cheng Li, Ni Zhao

**Affiliations:** ^1^ School of Electronic Science and Engineering Xiamen University Xiamen 361005 P. R. China; ^2^ Department of Electronic Engineering The Chinese University of Hong Kong Shatin New Territories Hong Kong SAR China; ^3^ Future Display Institute of Xiamen Xiamen 361005 P. R. China

**Keywords:** colloidal quantum dot, optical devices, optoelectronic devices, photonic structures

## Abstract

Colloidal quantum dot (QD), a solution‐processable nanoscale optoelectronic building block with well‐controlled light absorption and emission properties, has emerged as a promising material system capable of interacting with various photonic structures. Integrated QD/photonic structures have been successfully realized in many optical and optoelectronic devices, enabling enhanced performance and/or new functionalities. In this review, the recent advances in this research area are summarized. In particular, the use of four typical photonic structures, namely, diffraction gratings, resonance cavities, plasmonic structures, and photonic crystals, in modulating the light absorption (e.g., for solar cells and photodetectors) or light emission (e.g., for color converters, lasers, and light emitting diodes) properties of QD‐based devices is discussed. A brief overview of QD‐based passive devices for on‐chip photonic circuit integration is also presented to provide a holistic view on future opportunities for QD/photonic structure‐integrated optoelectronic systems.

## Graphical abstract

The recent advances of the integrated quantum dot (QD)/photonic structures in many optoelectronic and optical devices for performance enhancement and new functionalities are summarized in this review. The use of four typical photonic structures applied in either modulating the light absorption or light emission properties of QD‐based devices is discussed, and the innovative QD‐based on‐chip photonic circuit is briefly overviewed.

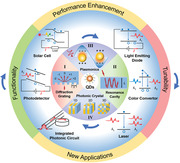

## 1 Introduction

There have been extensive research efforts in integrating photonic structures with low‐temperature processed semiconductors, such as organic semiconductors,^[^ ^1^ ^]^ colloidal quantum dots (QDs),^[^ ^2^, ^3^, ^4^ ^]^ organohalide perovskites,^[^ ^5^, ^6^, ^7^ ^]^ and 2D materials,^[^ ^8^ ^]^ aiming at achieving high‐performance optoelectronic or optical devices with tunable functions and low fabrication cost. Among the semiconductors, colloidal QD is a particularly promising material family for interaction with photonic structures due to its high photoluminescence (PL) quantum yield,^[^ ^9^ ^]^ widely tunable bandgap from ultraviolet (UV) to far‐wave infrared (IR),^[^ ^10^ ^]^ great photostability,^[^ ^3^ ^]^ and large surface‐to‐volume ratio that allows for various interface modification and functionalization. QDs have been exploited to form the active layer of a variety of optical and optoelectronic devices^[^ ^11^, ^12^, ^13^ ^]^ such as solar cells and infrared photodetectors that are based on lead chalcogenide or mercury chalcogenide QDs^[^ ^4^, ^14^, ^15^ ^]^ and light‐emitting diodes (LEDs), color convertors, and lasers that are based on cadmium chalcogenide or perovskite QDs.^[^ ^3^, ^6^, ^16^ ^]^ In order to enhance the light absorption/extraction properties or to tune the spectral characteristic of these QD‐based devices, researchers have developed various QD/photonic structure‐integrated device architectures and achieved significant performance enhancement and/or new device functions. The rapid progress in this research area has brought QD technologies one step closer to enable new‐generation electronic and photonic devices that are superior to the ones based on traditional semiconductor technologies.

**Figure 1:**
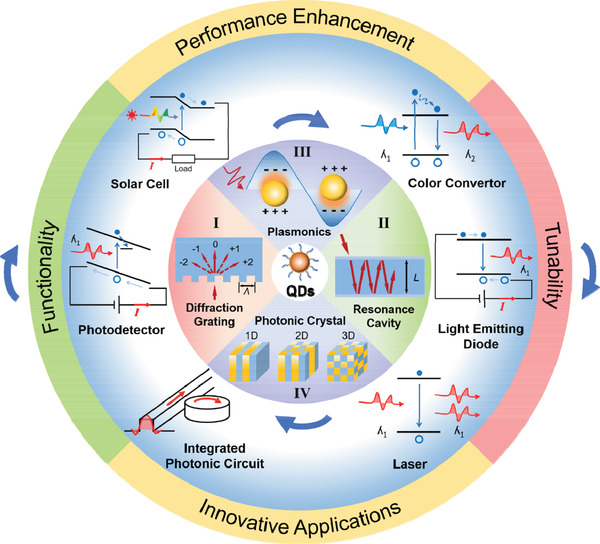
Schematic summary of the integration of photonic structures and colloidal quantum dots (QDs) for optoelectronic and on‐chip transmission devices. There are four typical photonic structures, namely, I) diffraction grating, II) resonance cavity, III) plasmonic structure, and IV) photonic crystal for light management, and they can be respectively applied to different devices. Besides performance enhancement, the photonic structures also allow for tuning of optical properties and functionalities, thus providing opportunities for new applications.

In this review, we summarize the recent advances of QD/photonic structure‐integrated optical and electronic devices following the schematic outline shown in **Figure** **1**. In Section 2, the basic optical properties of four typical photonic structures (viz. diffraction gratings, the resonance cavities, plasmonic structures, and the photonic crystals (PCs), which are used most often for light management of QD devices, are introduced. In Sections 3 and 4, the operation principles and the fabrication methods of integrating these photonic structures in QD devices are described according to different application scenarios, e.g., light absorption (solar cell and photodetector) and light emission (color converter, laser, and light‐emitting diode). Besides performance improvement, it is highlighted that the integration of photonic structures could offer new tunable properties and functionalities (e.g., selection of polarization and wavelength for photosensing, control of direction, polarization, wavelength and spontaneous emission rate for light emission, etc.). In addition, novel applications including hyperspectral or integrated detection, patternable light emission, and dual wavelength lasing are also presented. In Section 5, the emerging applications of QD‐based passive devices for on‐chip photonic circuit integration are briefly overviewed, providing a different perspective on the potential applications of QD‐integrated photonic structures. Finally, we evaluate the benefit of applying photonic structures in different types of QD devices and discuss the future challenges and opportunities in QD/photonic structure‐integrated devices.

## 2 Optical Properties of Photonic Structures

In this section, we briefly introduce the optical properties of the four photonic structures commonly used with QD‐based devices to illustrate the basic interaction theory between the photonic structures and the QDs for device performance optimization. The detail descriptions about the application examples and interaction models are provided in Sections 3 and 4.

### 2.1 Diffraction Grating

Figure 1I provides a schematic of a typical 1D diffraction grating. This structure is frequently adopted to facilitate the coupling‐in and coupling‐out of light in a wave‐guiding medium. Assuming that a ray of light in the air is incident normal to the grating medium of a refractive index *n* and the grooves are periodically spaced with a period *Λ*, from the phase‐matching requirement, the general expression for the diffraction angles of the forward diffracted light is given by

(1) iλ=nΛsinθ

where *i* is an integer number indicating the diffraction order, *λ* is the light wavelength, and *θ* is the angle of diffraction in the grating material of the *i*th‐order diffracted light.^[^ ^17^, ^18^ ^]^ Accordingly, a grating structure could be applied in QD‐based solar cells or photodetectors to increase the optical path of the incident light through diffraction and then coupling of the diffracted light to the waveguided modes supported by the QD layer to enhance light absorption. Reversibly, a grating structure can also be applied in QD‐based LED or color conversion layer to enhance the light outcoupling efficiency.

### 2.2 Resonance Cavity

Figure 1II presents a simplified model of a light ray path in a resonance cavity consisting of two reflective mirrors. This structure is frequently utilized to enhance the light absorption or emission over a narrow spectral range through constructive/destructive interference. Assuming that the cavity is placed in air and that light is normal incident onto the planar surface of the cavity medium of a refractive index *n*, the light, after reflected multiple times by the internal surfaces of the cavity, can constructively interfere with itself at a resonance condition

(2) $$2nL\cdot \left(2\pi /\lambda \right)=m\cdot 2\pi$$

where *m* is a positive integer number, *L* is the length of the cavity, and *λ* is the light wavelength. (Here, the phase changes at the reflection surfaces are neglected as the refractive index of the microcavity is normally larger than those of surrounding media in practical applications.)^[^ ^19^, ^20^ ^]^

A cavity structure can be conveniently introduced into QD‐based solar cells or photodetectors by utilizing the top and bottom electrodes of the devices as the mirror pair, which can enhance the light absorption of the QD layers within the cavity at a certain wavelength range. A cavity structure is also a critical component of lasers for its role in raising local optical density for efficient spontaneous emission of the gain media—QDs.

### 2.3 Plasmonic Structure

**Figure 2:**
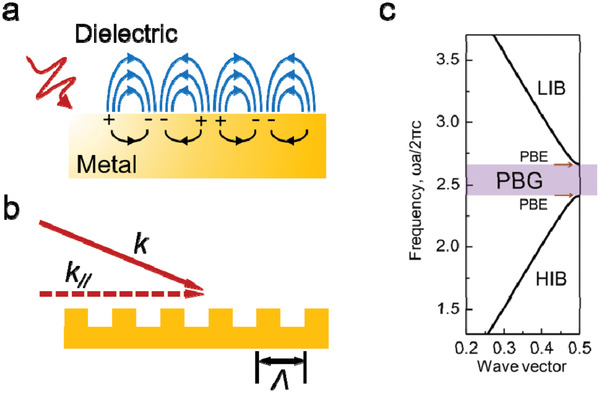
SPP and photonic bandgap. a) Schematic of the propagating surface plasmon polaritons (SPPs). b) Phase‐matching of the incident light to SPPs with periodic grating structure. c) Simulated photonic band structure of a typical 1D PC Bragg reflector. Reproduced with permission.^[^ ^21^ ^]^ Copyright 2014, The Royal Society of Chemistry.

Figure 1III illustrates generation of localized surface plasmon resonance (LSPR) through light‐excited oscillation of electrons at a metal nanoparticle (NP) surface—one of the most common plasmonic structures. When the particle size is comparable to or smaller than the excitation wavelength, the oscillated electromagnetic field triggers the oscillation of the electron cloud of the metal nanoparticle at the resonance frequency, displacing the cloud center away from its original position (i.e., the center of the positive charges).^[^ ^7^, ^22^ ^]^ The resonance frequency depends on the material type, size and shape of the nanoparticles, the effective electron mass, and the permittivity of the surrounding medium. Besides LSPR, surface plasmon can also be generated at the interface between a planer metal film and a dielectric layer. As shown in **Figure** **2**a, the propagating surface plasmon polaritons (SPPs) are generated by the collective oscillation of free electrons at the metal/dielectric interface and coupled to the guided transverse magnetic (TM) modes. The propagating SPPs have larger momentum than the radiative modes in the dielectric, which lead to evanescent decay on both sides of the interface. Therefore, a periodic grating structure is often created at the metal surface to scatter‐out the extra momenta and thus facilitate the excitation of SPPs with incident light or light radiation from the SPP modes. Considering a 1D grating with period *Λ* (as illustrated in Figure 2b), the phase‐matching condition is

(3) $$k_{\mathrm{spp}}=k_{//}\pm m\cdot \left(2\pi /\Lambda \right)$$

where *k*_spp_ is the wave vector of the SPPs, *k*_//_ is the in‐plane wave vector of incident/radiated light, and *m* is a positive integer.^[^ ^22^ ^]^

To integrate with the QD‐based devices, the LSPR is commonly enabled by embedding the plasmonic metal NPs in a QD layer or other functional layers, while the excitation of propagating SPP modes normally occurs at the metal electrode/adjacent layer interface. Both the LSPR and propagating SPPs have the ability to concentrate the near‐field light field and to enhance the far‐field light scattering in the vicinity of the metal structures, both leading to enhanced light absorption of the QD layers at plasmon resonance. Besides the optical effects, the metallic nature of plasmonic structures also makes them a tool to modify the electronic properties of the surrounding medium. As to be reviewed later, plasmonic structures are found to affect the exciton binding energy^[^ ^23^ ^]^ or Fermi level of the neighboring semiconductors.^[^ ^24^ ^]^ On the other hand, the localized density of optical states (LDOS) in the vicinity of the metal structures could be significantly enhanced with the plasmon resonance, thereby increasing the radiative rate of the emissive material like QDs surrounding the structures via Purcell effect.^[^ ^25^ ^]^ As such, in some plasmonic enhanced QD‐based light‐emitting devices, the radiative recombination process is dominated by the exciton–plasmon coupling induced with the strong interaction between the QDs and plasmonic structures.^[^ ^7^, ^26^ ^]^

### 2.4 Photonic Crystal

Figure 1IV provides schematic structures of PCs in 1D, 2D, and 3D, with different colors denoting the materials of different refractive indexes.^[^ ^27^, ^28^ ^]^ PCs are artificial micro/nanostructures formed with periodically arranged media of different refractive indices. With the energy of the propagated electromagnetic wave modes respectively concentrated in the high or low refractive index region, a photonic bandgap (PBG) is opened up in the PCs.^[^ ^29^ ^]^ Figure 2c illustrates the photonic band structure of a typical bulk 1D PC with alternatively stacked high and low refractive index layers.^[^ ^21^ ^]^ The bands above and below the PBG are respectively named as high refractive index band (HIB) and low refractive index band (LIB). The PBG forbid the light of certain wavelengths to propagate through the PCs and thus achieve a reflective surface with wavelength selectivity for device applications, which could be employed to form the high‐quality‐factor (high‐*Q*) reflection mirrors for QD‐based lasers. Besides the wavelength selectivity, another interesting feature of PCs is the nearly zero group velocity of the photonic band edge (PBE) modes, corresponding to a strong LDOS. Accordingly, by matching the PBE modes with the light pumping or emission wavelength of the QDs, the PCs can be adopted to modulate the light absorption and emission properties in QD‐based color conversion or lasing demonstrations. Also, the periodic surface of the PCs, which is similar to the diffraction gratings, could facilitate the light outcoupling from the QD films.

Considering the simplest case of PCs—a 1D PC, the PBG of the 1D PC can be understood as the result of Bragg reflection.^[^ ^28^, ^30^, ^31^ ^]^ The reflected waves from the periodic interface are in phase with each other, resulting a constructive interference to reject the propagation wave. Assuming that the propagated light perpendicularly enters a 1D PC (formed by two alternatively patterned layer materials with refractive indices *n*_1_, *n*_2_, and thickness *d*_1_, *d*_2_), the relation between the central reflected wavelength *λ*_0_ and the optical thickness of the PC can be evaluated as

(4) $$\left(\lambda_{0}/2\right)\cdot m=n_{1}\cdot d_{1}+n_{2}\cdot d_{2}$$

where *m* is a positive integer.^[^ ^30^, ^31^ ^]^ The width of the PBG depends on the refractive index contrast between *n*_1_ and *n*_2_. For 2D and 3D PCs, the derivations of the PBGs and the complex band structures are well‐summarized in ref. ^[^ ^29^ ^]^.

Based on Equations (1)–(4), it is apparent that the functional wavelength of the photonic structures is highly dependent on their dimensions/period. Therefore, the geometry of the photonic structures should be well designed to match with the specific bandgap of a target QD system.

## 3 Photonic Structure‐Enhanced QD‐Based Light‐Absorbing Devices

### 3.1 Solar Cells

Lead chalcogenide (e.g., lead sulfide (PbS)) QDs, with a high absorption coefficient and broad spectral response covering both the visible and infrared wavelength regimes, have shown potential to expand the energy‐harvesting spectrum of current solar cells.^[^ ^32^, ^33^, ^34^, ^35^ ^]^ However, due to the relatively low charge carrier mobility and high trap density of the QD layer, the carrier diffusion length is normally limited to ≈400 nm or lower,^[^ ^36^ ^]^ which is much thinner than the thickness (≈1 µm) required to fully absorb the infrared light.^[^ ^37^ ^]^ To overcome this limitation, a series of light management techniques exploiting photonic structures have been developed to enhance light absorption of the QD layer, and thereby to improve the external quantum efficiency (EQE) and power conversion efficiency (PCE) of the corresponding solar cells.

#### 3.1.1 Functional Layers with Textured or Grating Structures

**Figure 3:**
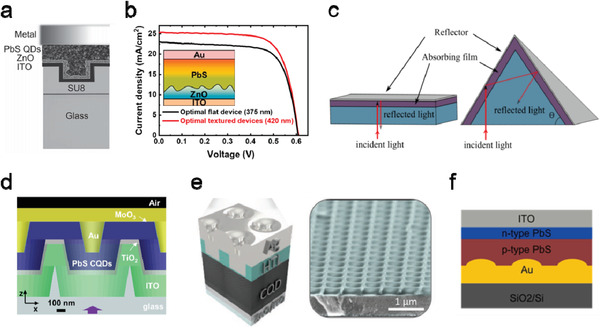
QD solar cells with textured or grating structures. a) Schematic of a PbS QD/ZnO heterojunction solar cell fabricated on imprinted electrodes. Reproduced with permission.^[^ ^41^ ^]^ Copyright 2013, Wiley‐VCH. b) The current–voltage characteristics comparison in the optimized flat and textured (inset) devices, measured under AM1.5 solar illumination. Reproduced with permission.^[^ ^42^ ^]^ Copyright 2019, American Chemical Society. c) Illustration of the light path enhancement in a pyramid‐patterned TiO_2_ layer of a PbS QD solar cell. Reproduced with permission.^[^ ^43^ ^]^ Copyright 2014, American Chemical Society. d) The schematic cross‐section of the 3D hexagonal nanostructured QD solar cell. Reproduced with permission.^[^ ^45^ ^]^ Copyright 2013, Springer Nature. e) (Left) Schematic of the QD devices with patterned hole transporting layer (HTL). (Right) Scanning electron microscopy (SEM) image of the nanoimprinted HTL. Reproduced with permission.^[^ ^37^ ^]^ Copyright 2019, Wiley‐VCH. f) Schematic of the photodiode structure with a periodic patterned SPP coupler. Reproduced with permission.^[^ ^48^ ^]^ Copyright 2014, American Chemical Society.

A QD‐based solar cell is typically a photodiode with a back‐illuminated multilayer structure consisting of, in sequence, a bottom transparent electrode, an electron transporting layer (ETL), a QD active layer, a hole transporting layer (HTL), and finally, a top metal electrode (the positions of the ETL and HTL can be switched in some cases). A series of research efforts were made to introduce textured structures in different layers of QD solar cells to enhance the light absorption or charge extraction and consequently to improve the EQE and PCE.^[^ ^38^, ^39^, ^40^, ^41^, ^42^, ^43^, ^44^, ^45^, ^46^, ^47^ ^]^ For example, Mihi et al. reported an efficient and controllable light trapping architecture in a PbS QD/ZnO heterojunction solar cell by patterning the back‐illuminated electrode and n‐type ETL.^[^ ^41^ ^]^ Using a soft‐nanoimprinting polydimethylsiloxane (PDMS) stamp, a layer of photoresist with 2D holes was first patterned on a glass substrate, followed by deposition of an indium tin oxide (ITO) layer to form a patterned electrode (**Figure** **3**a). Using light‐field simulation, the authors observed that the wavelength‐dependent absorption enhancement varied with the lattice parameter of the electrode pattern, indicating that the enhancement originated from the light diffraction of the square lattice and the consequent coupling of diffracted light to the trapping modes of the device. Compared to the planar device configuration, up to 100% EQE enhancement was achieved in the imprinted devices in the 600–1000 nm wavelength range, resulting in ≈18% increment in the short‐circuit current (*J*_sc_). Recently, Kim et al. pointed out that the Fresnel reflection at the PbS QD/ZnO interface caused the optical interference, so as to affect the light field distribution within the device and accordingly lower the optimal thickness of the active layer.^[^ ^42^ ^]^ To interrupt the light interference in the active layer, they fabricated a textured ZnO structure with <80 nm feature size to minimize the light scattering and realize the gradually changed effective refractive index across the adjacent layers. Such device structure increased the optimal thickness of the PbS QD layer to 420 nm, achieving a PCE of 11.1% (Figure 3b). The optical absorption of a QD solar cell can also be enhanced by light path folding through multireflection from micropatterned electrodes, as demonstrated in a PbS QD hierarchical solar cell with a microscale pyramid‐shaped TiO_2_ layer (Figure 3c).^[^ ^43^, ^44^ ^]^ Under this scheme, the absorption enhancement was dominated by geometric‐optics, and it was highly sensitive to the inclination of the pyramid angle. The PbS QD hierarchical solar cells are fabricated through a transfer‐stamping technique using anisotropic etched silicon wafers as the original moulds; with the optimal patterns an improvement of ≈24% in *J*_sc_ (compared to the planar cells) was achieved. Adachi et al. reported a 3D hexagonal nanostructured QD solar cell fabricated on a prepatterned glass substrate with a feature height of a few hundred nanometers (Figure 3d).^[^ ^45^ ^]^ 3D patterning was replicated in each layer of the device to achieve a broadband absorption enhancement in the 600–1100 nm range, leading to a 31% increment in *J*_sc_ in the 3D nanostructured devices as compared to the planar ones with an equal volume of the light absorbing material. The light absorption of QD solar cells can also be enhanced by embedding dielectric resonance spheres into the active QD layer,^[^ ^46^ ^]^ or by forming a nanostructured back‐mirror at the QD/top electrode interface.^[^ ^47^ ^]^ As such nanostructures may increase the surface recombination rate of the active layer, Baek et al. proposed to induce the nanostructured back‐mirror through a copolymer‐based imprint‐patterned HTL.^[^ ^37^ ^]^ In their design the HTL material was spun onto the flat QD layer and then imprinted by a prepatterned PDMS stamp from the top surface to form a 2D square array of cylinders with controllable depth; finally a thin electrode layer was conformally coated on the HTL surface (Figure 3e). This patterned photonic structure showed a pronounced wavelength‐selective property and demonstrated significant light absorption enhancement in the near‐infrared (NIR) range. The EQE of the imprinted device was as high as 86% at 1220 nm, suggesting its promising potential for tandem solar cell applications.

The aforementioned nanoscale periodic structures, when patterned into the metal electrodes, may modulate the propagating SPP modes at the metal/QD interface to further affect light absorption.^[^ ^37^, ^45^, ^47^ ^]^ To reveal the role of the SPP coupling in light absorption enhancement, Beck et al. developed a model to analyze the origin of the wavelength‐selective enhancement of grating structures.^[^ ^48^ ^]^ The model adopted a top‐illuminated PbS QD homojunction photodiode with a 2D patterned gold back electrode as the SPP coupler (Figure 3f), and the simulation results showed that the absorption enhancement occur at the coupling wavelengths when the propagation constants of the trapped propagated modes equal to the reciprocal lattice vectors of the SPP couplers. The spatial overlap of the mode profile with the grating was critical to excite a certain waveguiding mode.^[^ ^49^ ^]^

#### 3.1.2 Embedding Plasmonic Metal Nanoparticles

**Figure 4:**
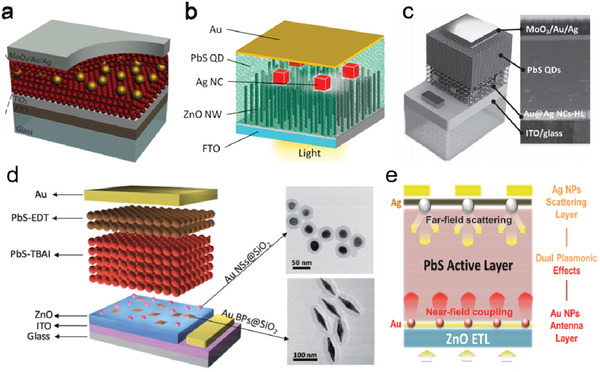
QD solar cells with embedded metal NPs. a) Schematic of the PbS QD heterojunction solar cell embedding with SiO_2_/Au nanoshells. Reproduced with permission.^[^ ^52^ ^]^ Copyright 2013, American Chemical Society. b) Schematic of PbS QD/ZnO nanowire bulk heterojunction solar cell with silver nanocubes (NCs). Reproduced with permission.^[^ ^54^ ^]^ Copyright 2015, American Chemical Society. c) Schematic of QDs solar cells with Au@Ag core–shell NCs in HTL, with the cross‐sectional TEM image of the device. Reproduced with permission.^[^ ^57^ ^]^ Copyright 2015, Wiley‐VCH. d) Schematic of PbS QD solar cells with the mixture of Au bipyramids (BPs)@SiO_2_ and Au nanospheres (NSs)@SiO_2_, and the TEM images of Au NSs@SiO_2_ and Au BPs@SiO_2_. Reproduced with permission.^[^ ^58^ ^]^ Copyright 2017, Wiley‐VCH. e) Cross‐sectional illustration of the PbS QD solar cells with the dual‐plasmonic effects of Au and Ag NPs. Reproduced with permission.^[^ ^59^ ^]^ Copyright 2020, Springer Nature.

LSPR, which is commonly enabled by embedding noble metal NPs in QD solar cells, is a widely employed strategy to enhance solar cell performance. From processing point of view, the LSPR strategy represents a more versatile approach to tune the optical properties of QD solar cells, as the tuning can be conveniently achieved by varying the material type, shape, size, and position of the NPs in the devices.^[^ ^11^ ^]^ The light‐trapping mechanisms associated with plasmonic metal NPs have been comprehensively summarized in a review of mesoporous solar cells,^[^ ^24^ ^]^ which highlights the prevail enhancement mechanisms in far‐field light scattering and near‐field focusing of light. For metal NP–QD systems, the plasmonic‐enhanced light absorption phenomenon was initially reported in nanocomposite films consisting of PbS QDs and gold nanospheres (NSs)^[^ ^50^, ^51^ ^]^ and soon after extensively studied in solar cell configurations. **Figure** **4**a illustrates the integration of SiO_2_/Au core–shell nanoparticles into PbS QD solar cell for plasmonic enhancement.^[^ ^52^ ^]^ The size of the SiO_2_/Au nanoshells was chosen such that the LSPR of the nanoshells occur at the excitonic peak of QDs, and the position of the nanoshells was controlled by drop‐casting the nanoparticles at different depths of the active layer. Compared to Au NSs and nanorods (NRs), SiO_2_/Au nanoshells have a larger scattering/absorption cross‐section ratio (*σ*_sca_/*σ*_abs_) and therefore induce stronger localized field resonance to enhance light absorption in the QD layer, leading to 35% photocurrent increment in the NIR range. A simulation model for plasmonic NP‐embedded QD solar cells was later developed for such type of device structures.^[^ ^53^ ^]^

Besides light‐field confinement, near‐field plasmon resonance of metal NPs may also tune the electronic properties of solar cell devices.^[^ ^24^ ^]^ For example, Kawawaki et al. reported a PbS QD/ZnO nanowire (NW) bulk‐heterojunction solar cell with Ag nanocubes (NCs) embedded in the QD layer (Figure 4b).^[^ ^54^ ^]^ It was found that the inclusion of the Ag NCs enhanced the EQE of the solar cell across its whole absorption range, even at the wavelengths where the optical absorption of the pure QD film already saturates. Such enhancement clearly suggested an improved exciton‐to‐electron conversion process and was explained by the plasmonic structure facilitating charge separation in the device. Kholmicheva et al. constructed a plasmonic nanocrystal solar cell by mixing core–shell Au/CdS NPs with core/shell (CS) PbS/CdS QDs to form the absorber layer of the solar cell, and demonstrated that the plasmon energy from Au NPs can be transferred to the bandgap transition of QDs and subsequently be used to generate photocurrent.^[^ ^55^ ^]^ Beck et al. showed that a Schottky nanojunction can be formed at the Ag NP–PbS QD interface; as such, the plasmonic Ag NP structure cannot only concentrate the light field but also induce a built‐in field to facilitate charge separation and enhance both the *J*_sc_ and *V*_oc_ of the device.^[^ ^56^ ^]^ Baek et al. embedded Au/Ag core–shell nanocubes (Au@Ag NCs) into the TiO_2_ layer of a PbS QD solar cell (Figure 4c).^[^ ^57^ ^]^ This hybrid design of the ETL not only enhanced light absorption of the solar cell, but also improved the carrier mobility of the TiO_2_ layer through trap‐filling with the electrons injected from the NCs. Both the *J*_sc_ and FF was optimized in the solar cells with the Au@Ag NCs, resulting a 21% increment in the PCE.

Recently, new strategies exploiting two kinds of plasmonic metal NPs to boost up the QD solar cell performances were demonstrated. Chen et al. simultaneously embedded Au/SiO_2_ core–shell nanobipyramids (BPs) and NSs at the interface between the ZnO layer and the PbS QD active layer of a solar cell(Figure 4d) and achieved a broadband EQE enhancement from the visible to NIR range, with the BPs contributing to the enhancement in the 400–1100 nm range and the NSs to the 300–750 nm range.^[^ ^58^ ^]^ Compared to the control devices without the embedded NPs, the dual‐NP embedded device exhibited improved *J*_sc_ and *V*_oc_, leading to a PCE increment from 8.09% to 9.58%. Another example is to place plasmonic NPs at two different locations of the device to utilize different plasmonic enhancement effects. Figure 4e illustrates a PbS QD solar cell structure containing dual‐metal (Ag and Au) NP layers.^[^ ^59^ ^]^ In this device, the photocurrent enhancement was achieved through a combination of far‐field scattering effect, dominated by the large Ag NPs (≈50 nm) at the HTL/top electrode interface, and near‐field plasmon resonance contributed by the small Au NPs (≈10 nm) at the ZnO ETL/QD interface.

#### 3.1.3 Structures with Cavity Resonance

**Figure 5:**
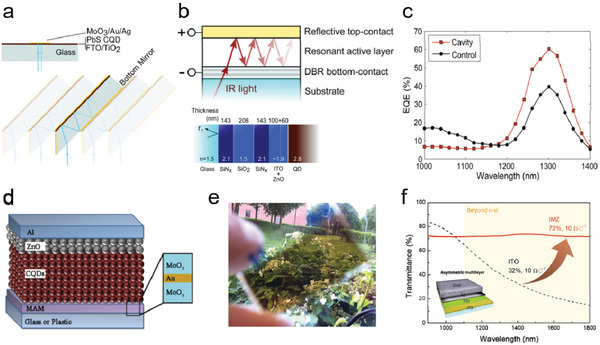
Cavity resonance and spacing layer. a) The normal QD solar cell with double pass of incident light (up) and the periodic arrangement of folded‐light‐path QD solar cell with multiple light passes (down). Reproduced with permission.^[^ ^60^ ^]^ Copyright 2013, Springer Nature. b) Schematic of the Fabry–Perot cavity IR solar cell (up) and the structure of the distributed Bragg reflector (DBR) mirror (down). c) Comparison in EQE spectrums of cavity‐based and control devices. Reproduced with permission.^[^ ^20^ ^]^ Copyright 2016, American Chemical Society. d) The schematic of the microcavity structured QD solar cell with MoO_3_/Au/MoO_3_ (MAM) electrode. Reproduced with permission.^[^ ^61^ ^]^ Copyright 2016, Elsevier Ltd. e) Photograph of the semitransparent QD solar cell with MAM electrode. Reproduced with permission.^[^ ^62^ ^]^ Copyright 2016, Wiley‐VCH. f) The infrared transmission comparison between the normal ITO and the asymmetric multilayer IMZ electrode (structure is shown in inset). Reproduced with permission.^[^ ^63^ ^]^ Copyright 2018, American Chemical Society.

As the QD solar cells are typically based on multilayered structures, the incident light is reflected at multiple interfaces during propagation, resulting in interference; under certain conditions, the interference may strongly affect the light field distribution within the device. **Figure** **5**a compares how light travels in a normal planar QD solar cell and in a folded‐light‐path (FLP) QD solar cell fabricated on a special fluorine‐doped tin oxide (FTO) glass substrate with a 45° cut edge.^[^ ^60^ ^]^ An optical cavity was formed between the metal electrode stacks of the FLP device, increasing the light reflection to six passes. As a result, the QD layer was able to effectively absorb all photons above the band edge, leading to enhanced photocurrent. Similarly, a Fabry–Perot (FP) resonance cavity formed in a QD solar cell can also enhance light absorption. By integrating a distributed Bragg reflector (DBR) mirror on the glass substrate, Ouellette et al. induced multiple reflection of light between the transparent DBR electrode and the back‐reflected metal electrode of a PbS QD solar cell (Figure 5b) and achieved NIR‐enhanced solar cell performance, with the EQE of the device reaching 60% at 1300 nm (Figure 5c).^[^ ^20^ ^]^ This feature is promising for tandem cell applications. Zhang and Johansson designed an inverted PbS QD solar cell microcavity structure by replacing the bottom ITO/FTO layer with a MoO_3_/Au(≈10 nm)/MoO_3_ (MAM) electrode for hole collection (Figure 5d).^[^ ^61^ ^]^ The use of the MAM electrode allows for formation of a microcavity between the MAM and the top metal electrodes. Furthermore, the light field distribution within the microcavity was optimized by tuning the thickness of the ZnO spacing layer, so as to maximize the light absorption within the QD layer. The MAM electrode was also adopted in a FTO/TiO_2_/PbS QD/MoO_3_/MAM solar cell structure to form a semitransparent light‐harvesting device for windows/building integrated photovoltaic applications (Figure 5e).^[^ ^62^ ^]^ Similarly, an asymmetric multilayer electrode structure was developed to maximize transmission of infrared light into the PbS QD layer of solar cells ((Figure 5f).^[^ ^63^ ^]^ By utilizing the FP cavity effect and optimizing the light field distribution, an EQE of 70% was reached at 1250 nm—the exitonic peak of the QDs.

### 3.2 Photodetectors

Heavy metal (cadmium, lead, mercury) chalcogenide QDs have shown great application potential in infrared photodetection at a wavelength range inaccessible by other solution‐processable semiconducting materials. For instance, photodetectors based on mercury chalcogenide QDs have shown photoresponse from short‐wave infrared (SWIR) and mid‐wave infrared (MWIR) range to long‐wave infrared (LWIR) range.^[^ ^10^, ^64^, ^65^ ^]^ Similar to solar cells, QD photodetectors also suffer from inefficient absorption in the IR range; therefore, integrating photonic structures in QD photodetectors is also deemed as an essential strategy to enhance the infrared light absorption of the photodetectors.^[^ ^2^, ^66^ ^]^

When designing photonic structures for QD photodetectors, there are a few factors to consider besides improvement of photocurrent (related to responsivity of photodetectors): first, the dark current level (related to noise level) and temporal response (related to 3 dB bandwidth) may be affected by some photonic structures, such as plasmonic NPs; second, for certain applications, the photodetectors are required to be wavelength‐selective or polarization‐selective, instead of responsive to broadband light; third, photodetectors can be achieved with various device platforms, such as photodiode, photoconductors, and phototransistors. Accordingly, in this section, we will first summarize the applications of photonic structures in the most commonly used photodiode‐type and photoconductive‐type QD photodetectors for enhancement of photosensitivity. Then, we will introduce some special cases where a photonic structure is integrated into a photodetector for achieving new functions (e.g., narrow‐band detection, polarization selection, etc.).

#### 3.2.1 Sensitivity Enhancement

**Figure 6:**
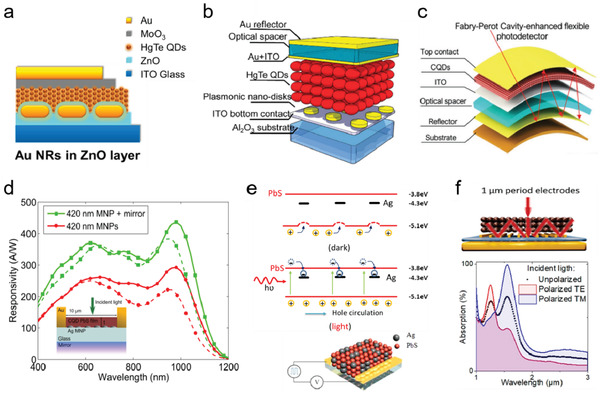
Sensitivity enhancement in photodetectors. a) Schematic of the NIR HgTe QDs/ZnO heterojunction photodiode detector with Au nanorods embedded in ZnO layer. Reproduced with permission.^[^ ^67^ ^]^ Copyright 2014, American Chemical Society. b) Illustration of the MIR HgTe QD photodiode detector with interference cavity and plasmonic disk array. Reproduced with permission.^[^ ^69^ ^]^ Copyright 2018, American Chemical Society. c) Schematic of FP cavity enhanced flexible HgTe QD detectors. Reproduced with permission.^[^ ^70^ ^]^ Copyright 2019, Wiley‐VCH. d) Responsivity of PbS QD photoconductor with and without back‐mirror, with (solid line) and without (dash line) metal NPs. Inset: Schematic of the photoconductor with Ag NPs and reflected mirror. Reproduced with permission.^[^ ^71^ ^]^ Copyright 2012, American Institute of Physics. e) Schematic of the PbS QD/Ag nanocrystal (NC) composite photodetector (down) and the mechanism of synergetic effect of Ag NCs for photosensing (up). Reproduced with permission.^[^ ^75^ ^]^ Copyright 2014, American Chemical Society. f) Schematics of the QD photoconductors fabricated with interdigitated electrodes designed to induce guide‐mode resonance (GMR) on a gold/SiO_2_ substrate and the corresponding simulated absorption spectra of incident light with different polarizations. Reproduced with permission.^[^ ^76^ ^]^ Copyright 2019, American Chemical Society.

For photodiode‐type QD photodetectors, both plasmonic structures and cavity structures are frequently adopted to enhance the sensitivity of the devices, as in the case of QD solar cells. Chen et al. demonstrated a plasmonic‐enhanced HgTe QD NIR photodetector adopting a basic photodiode structure of ITO electrode/ZnO ETL/HgTe QD layer/MoO_3_ HTL/Au electrode (**Figure** **6**a).^[^ ^67^ ^]^ The plasmonic structure was integrated into the device by performing twice sputtering of ZnO, between which a tape‐transfer process was applied to deposit randomly distributed and in‐plane oriented Au NRs onto the first ZnO layer. Such process is capable of placing the Au NRs at any depth in the ZnO ETL and therefore facilitates optimization of the optical and electronic properties of the device. The work demonstrated a 240% increment of *J*_sc_ in the plasmonic‐enhanced device, taking a device with identical layer thicknesses but without Au NRs as a reference. The work showed that by covering the Au NRs with an ultrathin ZnO layer, instead of placing the NRs in direct contact with QDs, one can minimize exciton quenching while still efficiently focusing light to the QD layer; also, the design does not raise the dark current level and response time. Ackerman, et al. utilized a cavity structure to enhance the MWIR absorption of a HgTe QD photodiode through constructive interference.^[^ ^68^ ^]^ Tang et al. further optimized the performance of such HgTe QD photodiodes by combining the cavity structure with an array of plasmonic gold nanodisks fabricated through electron beam (e‐beam) lithography.^[^ ^69^ ^]^ As shown in Figure 6b, the cavity was formed between the top and bottom electrodes. The top ITO optical spacer was used to maximize the reflection of light from the electrode to the QD layer, while the periodically patterned gold nanodisks (NDs) further optimized the MWIR absorption at the designed plasmonic resonance wavelength range. At a temperature of 85 K, the photodetector exhibited a maximum EQE of 45% at ≈4.5 µm and a detectivity of 4 × 10^11^ Jones, more than three times higher than the detectivity of the reference device that has no optical spacer and plasmonic structures. In a separate study, the research group has developed a flexible FP cavity‐enhanced SWIR HgTe QD photodetector.^[^ ^70^ ^]^ As illustrated in Figure 6c, the FP resonance was formed between the top semitransparent gold electrode and the bottom reflection mirror, with the photoresist as the optical spacer to control the resonance properties. A twofold enhancement in the photocurrent in SWIR wavelength range was observed and conserved under different bending angles.

Photoconductive‐type devices (including photoconductor and phototransistor) are another popular photosensing structure with the advantage of possessing high photoconductive gain. Unlike the vertical structures of photodiodes, photoconductive devices typically adopt planar structures with the light absorbing QD film covering a pair of prepatterned metal electrodes for current conduction. García De Arquer et al. reported a plasmonic‐enhanced PbS QD photoconductor where the QD layer was embedded with randomly distributed silver NPs at its bottom surface (Figure 6d).^[^ ^71^ ^]^ The strong scattering effect of the Ag NPs at LSPR facilitates the coupling of the incident light into the guided modes in the QD film. As a result, NIR light absorption of the Ag NR‐embedded device was significantly enhanced comparing to the control device without the NPs, and the photoresponse of the device was further increased by adding a silver mirror at the bottom of the substrate to double the light path. A series of similar plasmonic‐enhanced photoconductors were reported since then, including the Au nanocrystal‐enhanced perovskite QD photodetector,^[^ ^72^ ^]^ Au nanoantenna‐enhanced ZnO QD photodetector,^[^ ^73^ ^]^ and Au NP‐enhanced CuInSe_2_ QD phototransistor.^[^ ^74^ ^]^ Not only the optical absorption, the electronic properties of the photodetectors could also be optimized with the plasmonic structures. He et al. demonstrated a PbS QD/Ag NP composite photodetector by introducing the Ag NPs into the QD solution for co‐deposition.^[^ ^75^ ^]^ It was found that the presence of the Ag NPs in the device increased the photocurrent while at the same time reduced the dark current, both beneficial for increasing the detectivity of the device. As illustrated in Figure 6e, in dark, the Ag NPs caused the local band bending and hindered hole transport, resulting the reduced dark current; under light illumination, the Ag NPs serve as shallow traps of the photogenerated electrons and thus boosted the photoconductive gain, leading to increased photocurrent. In 2019, Chu et al. designed a QD photoconductor where a back‐reflected mirror and densely packed gold interdigitated electrodes were used together to induce guide‐mode resonance (GMR) within the QD layer (Figure 6f, up).^[^ ^76^ ^]^ On the one hand, the interdigitated electrodes can be considered as a diffraction grating to facilitate light coupling into the guide mode of the QD film to reach a near unity absorption (in TM polarization; Figure 6f, down). On the other hand, the short channel length also helped reduce the charge transit time between the electrodes to enhance the photoconductive gain. With the GMR structure, the responsivities of the PbS QD and HgTe QD photoconductors were enhanced by a factor of 250 (reaching an EQE of 86%) and 1000 (reaching an EQE of 342%), respectively, comparing to the reference devices without GMR structures at the desired wavelengths.

#### 3.2.2 Spectral‐ or Polarization‐Selective Photodetection

In practice, the desired properties of photodetectors are not limited to high responsivity or fast response, but also in tunability and functionality. In this regard, photonic structures provide an efficient way to manipulate the characteristic response of photodetectors for applications such as narrow‐band detection, polarization detection, and so on.

##### Narrow‐Band Detection

**Figure 7:**
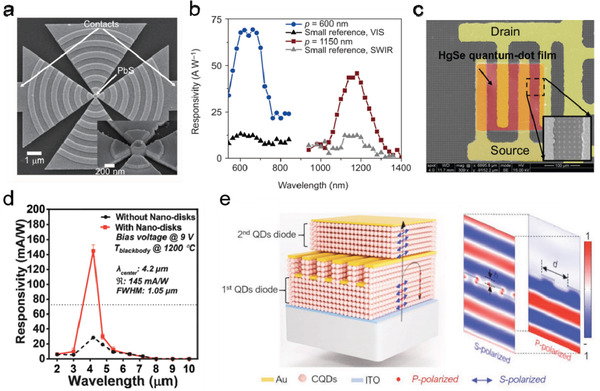
Narrow‐band and polarization‐sensitive QD photodetectors. a) The SEM image of a bull's eye structure (BES) photodetector with the inset highlighting the infiltration of the PbS QDs in the center between the Au triangles. b) Spectral responsivity of BES photodetectors with different pitches and their references without concentric arcs. a,b) Reproduced with permission.^[^ ^79^ ^]^ Copyright 2015, Springer Nature. c) The false color SEM image of the filterless narrow‐band infrared detectors consisting of transfer‐patterned HgSe QD film, interdigitated electrodes, and the patterned plasmonic disk arrays. d) The spectral responsivity of 4.2 µm samples with and without plasmonic disk arrays. c,d) Reproduced with permission.^[^ ^80^ ^]^ Copyright 2017, The Royal Society of Chemistry. e) Schematic of the structure and function of the two‐channel polarization detector with embedded bilayer wire grid polarizer and the simulated E‐field distribution passing through the device for s/p‐polarized light. Reproduced with permission.^[^ ^81^ ^]^ Copyright 2020, Wiley‐VCH.

Photodetectors with narrow‐band selectivity are desired in applications such as solar‐blind UV signal detection,^[^ ^77^ ^]^ filterless color discrimination in imaging systems.^[^ ^78^ ^]^ Diedenhofen et al. demonstrated a PbS QD photodetector containing a color‐tunable plasmonic bull's eye structure (BES) fabricated with e‐beam lithography.^[^ ^79^ ^]^ The BES shown in **Figure** **7**a, consisting of a nanohole surrounded by a concentric plasmonic grating structure, can efficiently couple the incident light into the SPP modes, guide the modes toward and constructively interference in the central aperture. By placing the PbS QDs in the central aperture as the photosensing material, a planar photoconductor enhanced by the nanofocusing lens was formed. Since the strong coupling would occur only when the reciprocal lattice vector of the grating matches with the propagated SPP modes, the nanofocusing effect is wavelength sensitive and tunable through the period of the grating (Figure 7b). Due to the very small amount of PbS QDs used to form the active layer, the noise level of the BES device was also greatly reduced comparing to the device with the QDs covering the whole arc grating area.

Plasmonic metal NPs can also be employed to realize narrow‐band detection. Tang et al. reported a plasmonic‐enhanced filterless narrowband HgSe QD MIR photodetector in a photoconductor structure where gold microdisks were periodically patterned between a pair of interdigitated electrodes (Figure 7c).^[^ ^80^ ^]^ By varying the radii of the disks, the plasmon resonance wavelength of the microdisks was adjusted from the MWIR to LWIR range, accompanied with sharp enhancement in the spectral response. When the plasmonic resonance wavelength of the microdisk matched well with the intraband absorption peak of the HgSe QDs, the narrowband detection of the HgSe QDs was optimized. As illustrated in Figure 7d , at the central response wavelength (4.2 µm) of the narrowband photodetector, the responsivity of the device was increased from 28.76 mA W^−1^ (without the disk array) to 145 mA W^−1^ (with the disk array), and the full‐width half‐maximum (FWHM) of the absorption peak was reduced from 1.84 µm (without the disk array) to 1.05 µm (with the disk array).

##### Polarization Detection

Photodetectors with anisotropic photoresponse to polarized light can simultaneously detect the intensity and polarization information of light, and therefore be utilized to improve photosensing resolution^[^ ^82^ ^]^ or achieve magneto‐optical data storage and processing.^[^ ^83^ ^]^ The plasmonic enhancement of the periodically patterned metal NPs can be designed to be polarization‐sensitive. Yifat et al. showed a MIR HgTe QD photoconductor containing a long‐striped optical nanoantenna (ONA) array patterned through e‐beam photolithography.^[^ ^84^ ^]^ The plasmonic enhanced photoresponse can only be observed when the polarization of the incident light was parallel to the antenna orientation due to the much stronger LSPR of the ONAs along the longitude direction. Recently, Tang et al. reported a HgTe QD‐based dual‐diode MIR photodetector with two‐channel‐polarization sensing ability.^[^ ^81^ ^]^ The authors first developed a pressure‐mediated nanoimprint technique that was capable of placing a quasi‐3D metallic nanostructure (such as grating, polarizer, and metal mesh) at a given depth of the HgTe QD active layer to achieve absorption enhancement or polarization‐ or wavelength‐selection. Figure 7e shows one example device—a two‐channel polarization detector in which a bilayer wire‐grid polarizer was embedded in the QD layer. Here, the metal wire‐grid not only served as the polarization selector, but also worked as the common electrode for the “back‐to‐back” packed top and bottom photodiodes. As such, the bottom diode (first QD diode in the figure) generates unpolarized information while the top diode (i.e., the second QD diode) responses to the polarization‐specific information.

##### Other New Functions

**Figure 8:**
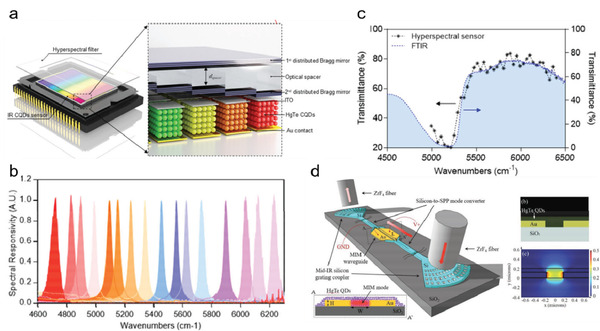
Photodetectors with novel functions. a) Schematic illustration of QDs hyperspectral sensor consisting of QDs sensor and hyperspectral filter. b) Measured spectral responsivity of fabricated QDs hyperspectral detectors. c) Measured transmittance spectra of water by (left axis) QDs hyperspectral detectors and (right axis) a commercial Fourier transform infrared (FTIR) spectrometer. a‐c) Reproduced with permission.^[^ ^85^ ^]^ Copyright 2019, Wiley‐VCH. d) The schematic of the on‐chip detection with the plasmonic–silicon hybrid waveguide system with the inset of the cross‐section of the metal–insulator–metal (MIM) waveguide with HgTe QD coating (left). The simulation schematic of the cross‐section of the HgTe QD‐loaded MIM waveguide and the electric field distribution of the MIM mode (right). Reproduced with permission.^[^ ^86^ ^]^ Copyright 2019, Wiley‐VCH.

Innovative functions, including hyperspectral sensing and on‐chip integrated sensor, have been demonstrated by integrating QD photodetectors with some specially designed optical systems. By integrating HgTe QD photodiodes with a DBR filter array, Tang et al. realized a hyperspectral sensor (a single detection unit with high spectral resolution) with 64‐channel spectral resolution in the SWIR range (**Figure** **8**a).^[^ ^85^ ^]^ Exploiting the strong FP resonance between the DBR mirrors, the DBR filter array offered narrowband spectral transmission and supported wavelength‐tuning through varying the optical spacer thickness. The SWIR hyperspectral sensor exhibited a spectral sensing window from 6500 to 4800 cm^−1^, with a FWHM of 40 +/− 7 cm^−1^ (Figure 8b). Promisingly, it showed comparable performance to a Fourier transform infrared (FTIR) spectrometer when used to measure the infrared transmission spectrum of water (Figure 8c). Besides of photodetection in free‐space, on‐chip photodetectors are promising in optical communication and photonic integrated circuit (PIC). Zhu et al. designed a plasmonic–silicon hybrid waveguide system integrated with a HgTe QD active layer and demonstrated on‐chip photodetection at the guided wavelength of ≈1600^[^ ^87^ ^]^ and ≈2300 nm, respectively (Figure 8d).^[^ ^86^ ^]^ The device was fabricated on the commonly used silicon‐on‐insulator substrate, using the input and output silicon waveguide grating coupler for light guiding. The center metal–insulator–metal (MIM) waveguide structure not only functioned as a plasmonic waveguide to concentrate the propagation mode into the QD region, but also served as the electrodes of the photodetector for current conduction. With this compact design the device showed a small footprint of 15 µm × 0.35 µm and a low noise equivalent power (NEP) of 8.7 × 10^−11^ W Hz^−1/2^, suitable for weak signal detection in silicon photonic circuits.

## 4 Photonic Structure‐Enhanced QD‐Based Light‐Emitting Devices

### 4.1 Color Conversion and Lasing

Due to their high PL efficiency and excellent color purity, QDs have long been researched for light‐emission related applications, e.g., color conversion units for display panels, white‐light sources, QD‐based lasers, optical amplifiers, and so on. Photonic structures have been employed in a series of strategies to realize light management of QD‐based PL devices. Here, we first summarize how different photonic structures are used to achieve color conversion and lasing modulation. Then, we will focus on some specific examples where photonic structures were used to the tune luminance properties such as direction, wavelength, and polarization. It is worth mentioning that this section does not cover regular QD lasers consisting of a QD gain medium and a resonance cavity,^[^ ^3^ ^]^ as the resonance cavity is an indispensable part of a basic laser structure. The discussions will be focused on how the optical properties of the QD medium are tuned through the use of novel photonic structures.

#### 4.1.1 Enhancement Mechanisms in Light Emission

##### Photonic crystal (PC)

As reviewed previously, PCs confine and control light propagation within their periodically modulated nanostructures. In 2007, Ganesh et al. coated CdSe/ZnS core–shell QDs on the surface of a 2D PC slab and observed significantly enhanced PL intensity from the QDs.^[^ ^88^ ^]^ By selecting the incident angle of the excitation light onto the PC slab, the leaky mode resonance can be generated right at the excitation wavelength, maximizing the absorption and PL efficiency of QDs. The nanostructured surface of the PC slab further facilitated Bragg scattering of emitted light to reduce light trapping of the guided modes. A similar mechanism was adopted to enhance the two‐photopump PL emission of CsPbBr_3_ perovskite QDs via a silicon 2D PC surface.^[^ ^89^ ^]^

**Figure 9:**
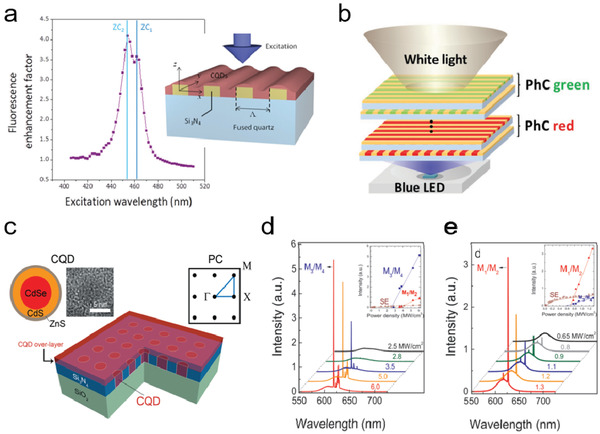
Color conversion and lasing demonstrations with photonic crystals (PCs). a) Fluorescence enhancement factor varied with the excitation wavelength of the planer 1D PC slab covering with QDs. Two PBE modes are marked with ZC_1_ and ZC_2_. Reproduced with permission.^[^ ^90^ ^]^ Copyright 2017, The Royal Society of Chemistry. b) Schematic of the stacked 1D PC layers coated with red and green QD emitters assembled with a blue LED chip for the white light generation. Reproduced with permission.^[^ ^91^ ^]^ Copyright 2018, Wiley‐VCH. c) Schematic of the 2D PC band‐edge laser coated with QDs. PL spectrums of the QD–PC lasers with various pump power densities for samples with QD coating thickness of d) 80 nm and e) 130 nm. Insets: The output light intensity of different modes versus the pump intensity. c‐e) Reproduced with permission.^[^ ^89^ ^]^ Copyright 2016, The Royal Society of Chemistry.

A planar QD‐coated PC slab can be readily used as a color conversion unit of a light source. **Figure** **9**a shows a planar 1D PC slab with the periodically spaced Si_3_N_4_ backbones covered with a CdSe/CdS/ZnS core–shell–shell QD layer.^[^ ^90^ ^]^ When the excitation wavelengths match the PBE modes, the fluorescence of the QD layer can be enhanced by more than fourfold. Using the PC design, the authors further demonstrated a compact and efficient white light source by stacking two kinds of 1D PC‐enhanced plates, respectively containing red and green QD emitters, on top of a blue LED chip (Figure 9b),^[^ ^91^ ^]^ and polarization insensitive color conversion slabs with 2D PC structures.^[^ ^92^ ^]^ A 2D PC can also be fabricated directly on the surface of a blue‐emitting InGaN/GaN quantum well (QW) structure using photolithography and dry etching processes, followed by coating of a CdSe/ZnS core–shell QD layer. The 2D PC can simultaneously enhanced the light extraction from the QDs and QW,^[^ ^93^ ^]^ and an efficient Förster resonance energy transfer (FRET) between the 2D PC patterned InGaN/GaN QW and CdSe/ZnS core–shell QDs was observed.^[^ ^94^ ^]^

PCs can also be adopted to modulate the radiative decay rate of QDs by changing the LDOS in their vicinity.^[^ ^95^, ^96^ ^]^According to Fermi's golden rule,^[^ ^97^ ^]^ by matching the emission wavelength of the QDs with the PBE modes, spontaneous emission could be boosted through the high LDOS at the PBE, an ideal condition for achieving lasing. Chang et al. observed dual band lasing in a CdSe/CdS/ZnS core–shell–shell QD layer‐coated 2D Si_3_N_4_ PC slab (Figure 9c).^[^ ^89^ ^]^ In this structure, the PBE modes, M_1_ and M_2_, were more concentrated in the dielectric Si_3_N_4_ backbone; while the PBE modes, M_3_ and M_4_, were more concentrated in the arrays filled with QDs. By tuning the thickness of the QD overlayer on the 2D PC, it is possible to tune the lasing threshold corresponding to different PBE modes and consequently select the output lasing wavelength (Figure 9d,e). The LDOS of a PC could be further enhanced by applying the PC to form a high‐*Q* resonance microcavity, and such type of structures have been used to achieve amplified spontaneous emission in a series of lasing demonstrations.^[^ ^98^, ^99^, ^100^ ^]^ The 1D and 2D PC slabs can also be fabricated with a cost‐effective way by nanoimprinting nanocavities in polymeric materials, and these PCs can be doped with CdSe/CdS core–shell QDs for PL enhancement and lasing applications.^[^ ^101^ ^]^

##### Plasmonic Metal NPs

**Figure 10:**
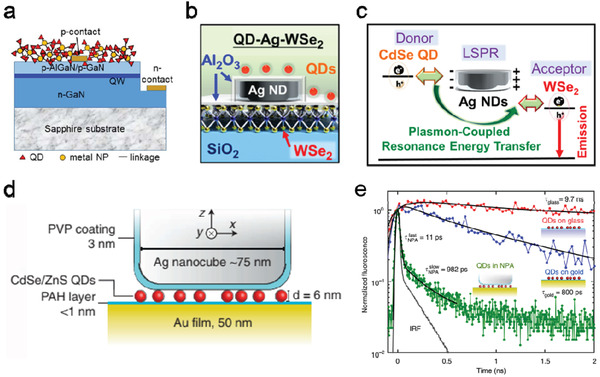
Color conversion and spontaneous emission enhancement with plasmonic structures. a) Illustration of a blue‐emitting quantum well (QW) LED covered with QD–metal NP composites on the top surface. Adapted with permission.^[^ ^109^ ^]^ Copyright 2019, Optical Society of America. Schematics of b) the QD–Ag NDs–WSe_2_ hybrid nanostructure for white light emission and c) the mechanism of plasmon‐coupled resonance energy transfer effect. b,c) Adapted with permission.^[^ ^111^ ^]^ Copyright 2020, American Chemical Society. d) The cross‐sectional schematic of the nanopatch antenna (NPA) consisting of an Ag nanocube and an Au film, spaced by a polyelectrolyte (PAH) layer and QDs. e) Comparison in normalized time‐resolved fluorescence (with exponential fittings in black line) of QDs on glass substrate, on Au film, coupling with an NPA, and the instrument response function (IRF). d,e) Adapted with permission.^[^ ^112^ ^]^ Copyright 2015, Springer Nature.

Integrating plasmonic metal NPs with QDs is another strategy to modulate the light emission properties of the QDs through affecting the LDOS associated with light absorption and emission processes. The exciton–plasmon interaction attributed significantly PL enhancement of QDs was observed and investigated in a series of QD–metal NP nanocomposite structures.^[^ ^102^, ^103^, ^104^, ^105^ ^]^ For example, Cai et al. respectively constructed the QD–Au NP and QD–Ag NP composites by linking the positively charged metal NPs to the negatively charged CdZnSeS/ZnS core–shell QDs^[^ ^106^ ^]^ and pointed out that the PL emission of the QDs could be improved either by matching the LSPR of the metal NPs with the excitation wavelength, or by matching the LSPR with the emission wavelength of the QDs. Similar to the QD‐coated PC color converters, the metal plasmonic structure–QD films can be applied in white light source. This idea was demonstrated by stacking the plasmonic enhanced QD‐based color conversion slabs on top of an InGaN/GaN‐based blue‐emitting LED,^[^ ^107^ ^]^ by placing Ag NPs into the interlayer between a QW LED and a QD emissive layer,^[^ ^108^ ^]^ or by directly coating a QD–metal NP composite film on top of a blue QW LED (**Figure** **10**a).^[^ ^109^ ^]^ They observed that the plasmonic metal structures could both enhance the QW emission and QD light absorption from the QW. Moreover, by simultaneously introducing Ag NPs and Au NPs into the color conversion mixture layer of green‐emitted phosphor and red‐emitted colloidal CdSe/ZnS QDs, the light emission from different materials could be both enhanced, so as to optimize the luminous efficiency and color rendering index.^[^ ^110^ ^]^

Beside the LSPR enhanced light absorption and emission, the plasmonic structure may facilitate the plasmon‐coupled resonance energy transfer between two light emission components to further improve the color conversion efficiency. Figure 10b is a hybrid nanostructure consisting of a 2D WSe_2_ monolayer and a core–shell CdSe/ZnS QD layer embedded with a plasmonic Ag ND array.^[^ ^111^ ^]^ By adjusting the diameter of the Ag NDs, the LSPR wavelength was finely tuned to overlap with the emission spectrum of the QDs and the absorption spectrum of the monolayer WSe_2_, and the significant plasmon‐coupled resonance energy transfer between the QDs and WSe_2_ was observed (Figure 10c). The hybrid structure exhibited a high downconversion color efficiency of 53%.

With their great efficiency in light‐field enhancement and capability of modifying LDOS, plasmonic nanocavities were introduced as a highly effective approach to reduce the spontaneous emission decay rate and enhance the emission intensity of QDs. Hoang et al. reported an ultrafast spontaneous emission light source with the plasmonic nanopatch antennas (NPAs), which is composed of a Ag nanocube coupling with an Au film spaced by the polymer layer and CdSe/ZnS core–shell QDs (Figure 10d).^[^ ^112^ ^]^ The spontaneous emission rate of the QDs under optical pump was boosted with the light reflected repeatedly between the plasmonic Ag nanocube and Au film. As shown in Figure 10e, the fluorescence lifetime of CdSe/ZnS QDs coupled with NPAs was pronouncedly decreased to less than 11 ps, which was corresponding to an enhancement factor of 880 in spontaneous emission rate and a 2300‐fold increment in fluorescence intensity comparing to the sample with QDs depositing on glass substrate. With the similar structure, a monolayer of PbS QDs was sandwiched in the gap region, and reported up to 500‐fold enhancement of PL intensity in near‐IR range.^[^ ^113^ ^]^

#### 4.1.2 Tunability in Direction, Polarization, and Emission Spectrum

##### Bull's eye structures (BESs)

**Figure 11:**
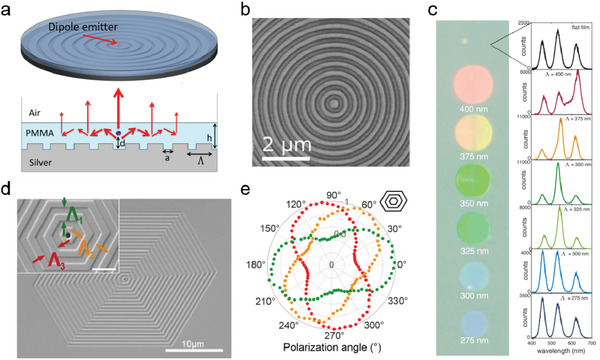
Luminance control with bull's eye gratings. a) (Up) Schematic of the hybrid metal–dielectric nanoantenna with a single QD dipole emitter (red sphere) in the center of the dielectric waveguide layer. (Down) Schematic of the cross‐section of this structure with the emission light paths (red arrow). Reproduced with permission.^[^ ^114^ ^]^ Copyright 2015, American Chemical Society. b) SEM image of the QD film directly patterned into bull's eye grating. c) The fluorescence images of bull's eye gratings with different pitches fabricated on mixed red–green–blue (RGB) QD films and the corresponding emission spectra. b,c) Reproduced with permission.^[^ ^116^ ^]^ Copyright 2017, American Chemical Society. d) SEM images of a hexagonal multiresonant antenna with the inset showing the large magnification of the central area. The period *Λ* varied in three directions. e) Polarization dependent fluorescent intensity of the hexagonal antennas filled with green‐ or orange‐ or red‐emitted QDs. d,e) Reproduced with permission.^[^ ^117^ ^]^ Copyright 2017, American Chemical Society.

A circular grating structure, also referred as BES, can be readily integrated with QD films to improve the directional extraction of QD emission without significantly increasing the complexity of the device structure. **Figure** **11**a shows a hybrid metal–dielectric nanoantenna consisting of a single CdTe/ZnS core–shell QD emitter in the center and a surrounding silver BES covered by a polymethacrylate (PMMA) waveguide layer.^[^ ^114^ ^]^ The light emitted from the CdTe/ZnS QD is first guided in the radial direction inside the dielectric layer and subsequently scattered out to the normal direction by the BES. Through the plasmonic mode interference, a strong light field confinement was realized in the center of the BES for efficient directional emission; as such, the hybrid metal–dielectric nanoantenna was used to direct the emission of the QD into a single‐mode fiber and achieve a maximal collection efficiency over 25% without the use of any collimator. The similar BES was adopted for laser applications. Gao et al. reported a highly directional and spatially coherent single‐mode surface‐emitting distributed feedback laser by coating a densely packed QD layer on a concentric circular Bragg grating.^[^ ^115^ ^]^

BES can also be adopted to control the spectrum and polarization of the PL emission of QDs. Prins et al. presented a template‐stripping method to directly pattern mixed red–green–blue (RGB) QD films into a high quality BES for luminance color control (Figure 11b).^[^ ^116^ ^]^ As shown in Figure 11c, by tuning the grating pitches, the BES could enhance the emission at specified wavelengths, resulting in the tunable emission colors. Moreover, the outcoupling efficiency of the spontaneous emission in the nanostructured QD layer was enhanced by up to sixfold as compared to an unstructured layer, and the strong directional beaming of lasing was observed. The research group further designed a new concentric grating structure to achieve polarization control of QD emission in different colors.^[^ ^117^ ^]^ The structure consisted of a central nanoaperture and concentric polygonal corrugations with varied period in each axis of the polygon (Figure 11d). This structure enabled independent control of individual color emission through phase‐matching in different directions. By filling the QDs with different emission wavelengths in the central nanoaperture, the polarization dependent fluorescence of different colors (green, orange, and red) was observed over the nanograting (Figure 11e), indicating that the spectral information could be coded into the polarization state of the QD emission for communication applications.

##### Plasmonic NP Lattices

**Figure 12:**
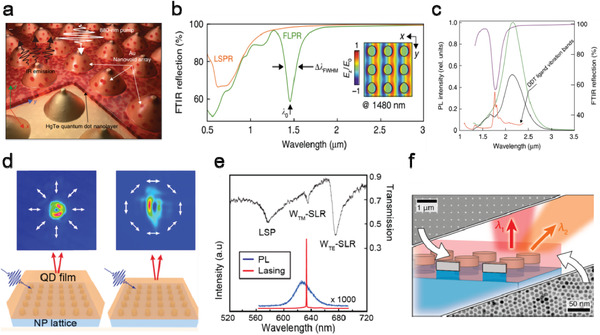
Luminance control with plasmonic NP lattice. a) Schematic of the laser‐printed Au nanobump arrays coating with HgTe QD layer for infrared emission. b) FTIR reflection spectrum of the plasmonic nanobump array with the FLPR referring to the first‐order lattice plasmon resonance. The LSPR of the isolated nanobump is also shown. The inset is the simulated E‐field distribution in *z*‐component at 1480 nm. c) Emission spectra of the HgTe QDs coating on a reference Si substrate (black) and an Au nanobump array (green). The normalized FTIR reflection spectrum of the Au nanobump array coated with QDs (purple) and the near‐IR absorption of the dodecanethiol (DDT) ligands of HgTe QD (red) are also shown. a‐c) Adapted with permission.^[^ ^119^ ^]^ Copyright 2020, Springer Nature. d) Schematic of QD–plasmon laser consisting of Ag NP lattice coating QD layer on top with varied thicknesses and the corresponding far‐field emission beams in radical or azimuthal polarization. e) Lasing (red) observed where the QD PL spectrum (blue) overlapped with the hybrid waveguide‐surface lattice resonance (W‐SLR) of the composite structure (black).d,e) Adapted with permission.^[^ ^120^ ^]^ Copyright 2020, American Chemical Society. f) Illustration of dual‐wavelength lasing with varied directions in a QD–plasmonic lattice laser. Insets are the SEM images of the plasmonic Ag ND lattice and the QDs coating on top. Adapted with permission.^[^ ^121^ ^]^ Copyright 2020, American Chemical Society.

Recently, the properties of periodically patterned plasmonic NP lattices were explored and their applications in tailoring the polarization, direction, and spectral properties of QD light emission were demonstrated. It was found that the in‐plane‐propagating waves diffracted by the ordered metal NP lattice could couple with the LSPR of individual metal NPs, resulting in drastic narrowing of the plasmonic resonances, and these resonances are named as plasmonic surface lattice resonances (SLRs).^[^ ^118^ ^]^ Compared with LSPRs, SLRs can couple with QD emission with stronger resonance and over a narrower spectral range and achieve higher quality factors. For instance, Sergeev et al. coated HgTe QDs on the surface of a femtosecond laser‐printed Au nanobump arrays (**Figure** **12**a) and demonstrated versatile control over the emission peaks of the QDs.^[^ ^119^ ^]^ The FTIR reflection spectra in Figure 12b denote the coupling between the LSPR of the single Au nanobump and the first‐order lattice plasmon resonance (FLPR) of the Au nanobump array. The near‐to‐mid IR emission of HgTe QDs was plasmonically enhanced via 1) wavelength matching between the LSPR and excitation light and 2) spectral overlap between the FLPR and QD emission. Moreover, the nonradiative decay loss of the QDs was significantly reduced through spectral matching between the FLPR and the vibration band of the QD ligands, allowing for optimization in both spectral distribution and PL intensity (Figure 12c).

A QD–plasmon laser with controlled polarization patterns was demonstrated with a Ag NP lattice covered with a layer of CdSe/CdS core–shell QDs (Figure 12d).^[^ ^120^ ^]^ Here, a waveguiding stack formed by the silica substrate, the QD layer and air was considered, and accordingly, waveguide‐surface lattice resonance (W_TM_‐SLR or W_TE_‐SLR) is formed through hybridization of the surface plasmons with the transverse electric (TE) or TM waveguide modes. When the PL emission of the QDs matches well with the W‐SLR modes, lasing occurs (Figure 12e), and it can be selected to be radially polarized or azimuthally polarized by varying the thickness of the QD layer and the polarization of the excited light. When the QD layer is sufficiently thick, higher‐order waveguide modes can coexist with the fundamental modes in the QD film. As shown in Figure 12f, a QD–plasmonic lattice laser was constructed by embedding a Ag ND lattice in the QD layer of an epoxy/QD/epoxy stack.^[^ ^121^ ^]^ Two lasing emissions associated with the TE_0_ and TE_1_ modes were demonstrated and optimized in different directions.

### 4.2 Light‐Emitting Diode

QD‐based light emitting diode (QLED) has been regarded as a highly promising candidate for next‐generation lighting and display systems, owing to the superior properties of QDs such as size‐tunable emission wavelength, bright and narrow‐band emission, and operation stability.^[^ ^122^ ^]^ The electroluminescence (EL) efficiency of QLEDs are mainly limited by two factors. First, the electron‐to‐photon conversion process could be hampered by low carrier mobility, inefficient charge injection, and quenching of excitons. Second, light extraction can be limited by total internal reflection due to the relatively high refractive index of the QD layer. Incorporating photonic structures in an LED has been a popular approach to boost the performances of various LEDs,^[^ ^7^, ^123^, ^124^ ^]^ and for QLEDs, this approach has been found to affect both the electronic and optical properties of the devices. In this section, we will first summarize the applications of the photonic structures in QLEDs for EL enhancement. Then, we discuss some special cases where a photonic structure is integrated into a QLED for achieving new functions such as artificial metamaterial surface and lasing with LED‐like devices.

#### 4.2.1 EL Enhancement

Comparing to the abundant research work in photonic structure‐enhanced QD solar cells and photodetectors, there have been less reports in light management strategies for QLEDs. So far, integration of surface plasmon metal nanostructures in QLEDs has been the most frequently adopted approach to achieve enhanced the EL of the devices,^[^ ^124^ ^]^ due possibly to its simple fabrication process and capability of tuning both the optical and electrical properties of a device. The plasmonic enhancement can be realized by i) embedding plasmonic metal NPs into the diode structure to enhance the internal quantum efficiency (IQE) of the device through exciton–LSPR coupling or ii) patterning the metal electrode to create corrugations at the metal/dielectric interface to facilitate outcoupling of SPP and waveguiding modes. The first approach has been demonstrated in multiple studies on plasmonic‐enhanced QLEDs, while for the second approach there has only been one report on a QLED with a patterned back‐reflected metal electrode serving as a nanograting to enhance the luminescence intensity.^[^ ^125^ ^]^

**Figure 13:**
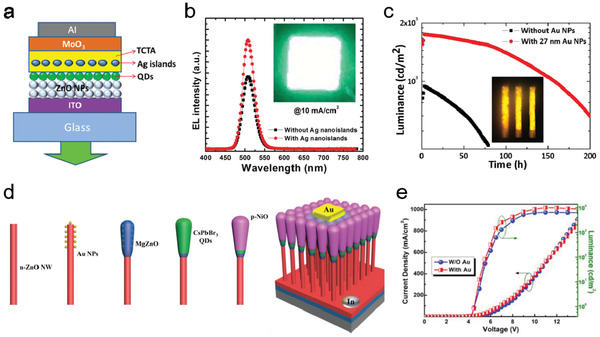
EL enhancement via photonic structures. a) Schematic of the device structure of the QLEDs embedded with Ag nanoislands. b) The comparison in EL spectra of the QLED with and without Ag nanoislands. Inset: The photographic image of the plasmonic QLED. a,b) Reproduced with permission.^[^ ^26^ ^]^ Copyright 2015, Wiley‐VCH. c) Luminance lifetime comparison between the plasmonic QLED with Au NPs and the control device, both operating in ambient condition at room temperature. Inset: EL picture of the plasmonic device. Reproduced with permission.^[^ ^126^ ^]^ Copyright 2016, Optical Society of America. d) Schematics of the preparation procedures of plasmonic enhanced CsPbBr_3_ QD‐based coaxial core/shell nanowire heterojunction architecture and the corresponding LED device. e) Comparisons in current density and luminance versus bias voltage of the devices in panel (d) with and without Au NPs decoration. d,e) Adapted with permission.^[^ ^127^ ^]^ Copyright 2018, Wiley‐VCH.

Similar to the case of plasmonic structure‐enhanced QD thin films (reviewed in Section 4.1.1), in QLEDs the radiative transition rate of QDs can be greatly enhanced through strong exciton–plasmon coupling in the vicinity of the plasmonic metal nanostructures, especially at the surface plasmon resonance wavelength. Therefore, the EL enhancement is sensitive to the distance between the metal NPs and QDs as well as the spectral overlap between their resonance and emission peaks. Considering that the direct contact between QDs and metal NPs may cause interfacial charge transfer and exciton energy transfer to surface plasmons and reduce EL,^[^ ^128^ ^]^ most plasmonic‐enhanced QLEDs are constructed by embedding plasmonic metal NPs into the adjacent layers of the QD emissive layer. **Figure** **13**a shows a plasmonic‐enhanced CdSe/ZnS core–shell QLED with Ag NPs embedded in the organic hole‐injection layer (HIL).^[^ ^26^ ^]^ The optimized device exhibited greatly enhanced green EL emission without any spectral change (Figure 13b). The maximum EQE and current density (CE) of the plasmonic QLED reached to 7.11% and 19.2 cd A^−1^, respectively, much higher than the EQE of 4.86% and CE of 13.2 cd A^−1^ for the control device without the Ag NPs. The average lifetime of the excitons in the QD film was shortened in the Ag NP‐embedded structure, which was explained by the strong plasmon–exciton coupling enhancing the radiative recombination channel. The type of device improvement has been reported in other plasmonic‐enhanced QLEDs, such as CdSe/ZnS QLEDs^[^ ^129^ ^]^ and perovskite QLEDs with embedded Au NPs.^[^ ^130^ ^]^

Besides enhancing EL intensity, metal plasmonic structures were also reported to suppress the efficiency roll‐off and improve the operation lifetime of QLEDs. Ji et al. incorporated Au NPs into the HIL of a green‐ and a red‐emitting QLED, respectively,^[^ ^131^ ^]^ and performed a comparative study on the “band matched” (i.e., emission band matched with the LSPR band) green QLED and “band mismatched” red QLED. The study highlighted the LSPR‐induced near‐field effects on EQE enhancement and delayed efficiency roll‐off and proposed that the auger recombination in the device can be suppressed by increasing the radiative rate of the QDs through plasmon–exciton coupling. Pan et al. reported a plasmonic enhanced yellow‐emitting CdSe/ZnS QLED with Au NPs sandwiched between the poly (ethylenedioxythiophene): polystyrenesulphonate (PEDOT:PSS) HIL and poly‐poly [(9,9‐dioctylfluorenyl‐2,7‐diyl) ‐co‐ (4,49‐ (N‐(4‐sec‐butylphenyl)) diphenylamine)] (TFB) HTL.^[^ ^126^ ^]^ They observed that the operation lifetime of the unencapsulated device in ambient conditions was significantly increased from ≈50 to ≈150 h after embedding the plasmonic structure in the QLED (Figure 13c) and also attributed the stability improvement to reduced nonradiative recombination.

Plasmonic metal NPs have also been incorporated into nonplaner LEDs. Shi et al. demonstrated a CsPbBr_3_ QD‐based coaxial CS heterojunction LED.^[^ ^127^ ^]^ As shown in Figure 13d, the coaxial CS heterojunction LED was formed by, from bottom up, first decorating an array of ZnO nanowires with Au NPs and then depositing in sequence a MgZnO spacer layer, a CsPbBr_3_ QD emissive layer, and a p‐doped NiO HTL. For the device embedded with Au NPs, a significant EL intensity enhancement was observed, with the luminance increasing from 6435 to 10 206 cd m^−2^ at an injection current density of 490 mA cm^−2^, while the current–voltage characteristics were only slightly affected (Figure 13e). Owing to the efficient light outcoupling, the good heat transfer property of ZnO nanowires, and the improved encapsulation of QDs provided by the coaxial structure, the device continuously operated for 60 h while only showing a decay of ≈14.3% in luminescence.

#### 4.2.2 Novel Electroluminescence Devices

##### Active EL Metamaterials

**Figure 14:**
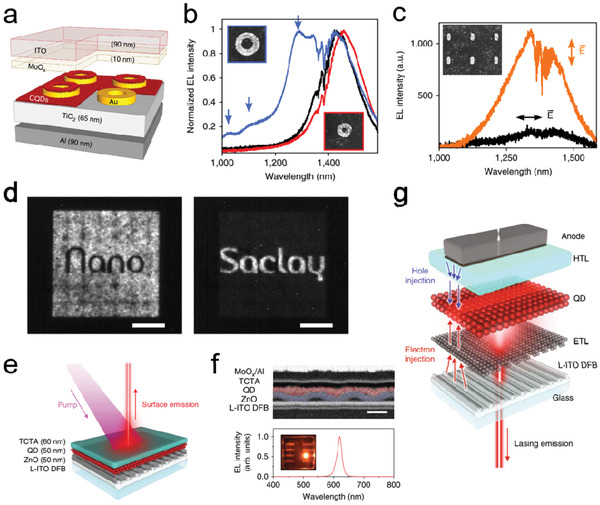
Active EL metamaterials and lasing with LED‐like device. a) Schematic of the metamaterial QLED. b) Comparisons in EL spectra of the reference QLED (black), the metamaterial‐based LED with small (red) and large (blue) Au rings. c) The EL spectra of a metamaterial QLED embedded with gold NR arrays in the parallel or perpendicular polarization directions to the long axis of the NRs. Inset: SEM of the NR array. d) Infrared images of the metamaterial LED with a linear polarizer aligned with the vertical (“Nano”) and horizontal (“Saclay”) set of NRs. Scale bars: 30 µm. a‐d) Adapted with permission.^[^ ^133^ ^]^ Copyright 2016, Springer Nature. e) Schematic of the optically pumped multilayered DFB laser employing the device structure as a typical p–i–n QD‐LED. f) The cross‐sectional SEM image of the EL device (scale bar: 200 nm) and the EL spectrum with the photograph of an operating device. g) A proposed quantum dot laser diode (QLD) architecture. e‐g) Adapted with permission.^[^ ^134^ ^]^ Copyright 2020, Springer Nature.

Metamaterials are artificial composites of metal and dielectric with the feature sizes comparable to the wavelength of the propagating electromagnetic wave, and they are frequently utilized to interact or modulate property of the wave.^[^ ^132^ ^]^ Le‐Van et al. reported an EL metamaterial based on a QLED architecture with a thin layer of QD coating on top of the patterned Au nanostructures (spaced with long‐chain ligands).^[^ ^133^ ^]^ With e‐beam lithography, Au NPs with varied shapes were periodically patterned in between the TiO_2_ ETL and PbS QD layer (**Figure** **14**a). Because of the strong exciton–plasmon coupling, nearly all of the EL‐relevant properties, including the brightness, spectral response, and polarization, can be modified by the LSPR of the Au nanostructures, as shown in Figure 14b. In particular, when Au NRs were used to form the plasmonic pattern, the EL emission of the device became polarization‐sensitive, which was determined by the alignment direction of the NRs (Figure 14c). With two different aligned sets of orthogonal Au NRs patterning on the same surface, this EL metamaterial device showed completely different EL images in different polarization directions (Figure 14d). This structure exhibited a discrete artificial EL pattern with subwavelength pixels, which may offer opportunities in structuring lighting in nanoscale.

##### Lasing with LED‐Like Device

By integrating a second‐order distributed feedback (DFB) resonator into a typical p–i–n LED architecture and using a low‐refractive‐index ITO (L‐ITO) as the cathode, an optically pumped low‐threshold laser was demonstrated (Figure 14e).^[^ ^134^ ^]^ The DFB resonating cavity was a 1D grating fabricated on the L‐ITO layer with its period matching the lasing wavelength; accordingly, single‐mode light amplification in the lateral direction was obtained. By fine tuning the thicknesses of the QD layer and the HTL to optimize the mode confinement, the authors realized optically pumped single‐mode lasing in the QD layer and demonstrated the structure can work as an LED (Figure 14f). This device can theoretically be driven as an electrically pumped laser (Figure 14g); however, the current density of the device was still much lower than the threshold required to reach the optical gain for stimulated emission.

## 5 Emerging QD‐Based Devices for Photonic Integrated‐Circuit Applications

There have been increasing research interests in QD‐based photonic structures and optical systems for on‐chip PIC applications. PIC is an integrated optical system consisting of a series of photonic components fabricated on a chip to precisely control and manipulate photons at micro‐ and nanoscale.^[^ ^135^ ^]^ Typical optical components in PICs include light sources, waveguides, grating couplers, optical processers (e.g., splitters, polarizers, amplifiers, modulators, and so on), and detectors. Traditional PICs are normally based on silicon technologies, but the low optical gain in these systems limit the active component applications. On the other hand, colloidal QDs are regarded as a highly promising class of gain materials for on‐chip active components such as optical amplifier^[^ ^136^, ^137^ ^]^ and coherent light source.^[^ ^3^, ^135^, ^138^ ^]^ However, the on‐chip integration of the colloidal QD active components with other functional photonic devices is still a challenge due to the complexity in processing different types of materials.

**Figure 15:**
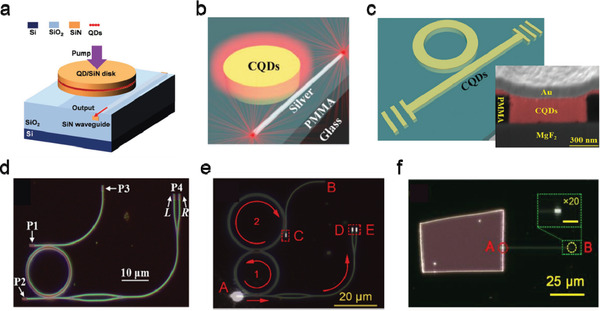
QD‐based photonic integrated circuit. a) Schematic of a vertical coupling configuration of a SiN/QD/SiN microdisk laser and a SiN bus waveguide. Adapted with permission.^[^ ^140^ ^]^ Copyright 2017, Wiley‐VCH. b) Schematic of a QD microcavity hybridized with a silver nanowire in tangential coupling. Adapted with permission.^[^ ^142^ ^]^ Copyright 2018, Wiley‐VCH. c) Schematic of a QD waveguide‐ring resonator (WRR) laser tangential coupling with a strip QD waveguide with the inset showing the cross‐sectional SEM image of the QD WRR. d) Dark‐field optical image of a complicated coupling structure with one WRR laser, one Mach–Zehnder interferometer, one Y‐splitter, two straight waveguides, two bending waveguides, and five gratings. c,d) Adapted with permission.^[^ ^143^ ^]^ Copyright 2019, The Royal Society of Chemistry. e) Optical images of the low‐loss QD‐based passive nanophotonic circuit when the incident light is focused at the grating A, with the red dashed rectangles indicating the scattered light from the decoupling gratings. f) Optical images of an integrated structure with a red QD microplate laser and a green QD waveguide (between A and B) under pumping lasing. e,f) Adapted with permission.^[^ ^144^ ^]^ Copyright 2020, Optical Society of America.

One effective strategy is to directly integrate the processing steps of colloidal QDs into the standard complementary metal oxide semiconductor (CMOS) manufacturing process for inorganic PIC components. Xie et al. fabricated a hybrid waveguide wire with a monolayer of CdSe/CdS QDs sandwiched between SiN layers deposited via plasma enhanced chemical vapor deposition (PECVD). After deposition of the top SiN layer, the PL peak of the QDs was preserved and a low transmission loss of 2.69 dB cm^−1^ was observed at the wavelength range beyond the absorption edge of the QDs. With the similar SiN/QD/SiN structure, the authors further demonstrated a free‐standing microdisk resonator^[^ ^139^ ^]^ and a microdisk multimode laser,^[^ ^140^ ^]^ both of which were vertically coupled with an on‐chip SiN bus waveguide. As illustrated in **Figure** **15**a, the light emission of the QDs was efficiently coupled with the whispering gallery modes (WGMs) of the microdisk and could access the outer PICs through the low‐loss SiN waveguide. Using e‐beam lithography and reactive ion etching (RIE), a DFB grating was directly fabricated on the surface of a SiN/QD/SiN waveguide and an on‐chip QD‐based single mode laser was demonstrated.^[^ ^141^ ^]^ Jung et al. demonstrated light outcoupling from a QD‐ PC band‐edge laser to a coplanar waveguide and grating outcouplers.^[^ ^100^ ^]^ Single‐mode lasing was generated by the QD–PC laser, and coupled into the adjacent passive waveguides and the grating outcouplers with a high efficiency as the components were fabricated on the same Si_3_N_4_ backbone slab.

In some cases, standard CMOS fabrication methods may bring damage to QD materials.^[^ ^145^ ^]^ To address this issue, an integration strategy for QD‐based WGM on‐chip laser and subwavelength plasmonic waveguides was proposed.^[^ ^142^ ^]^ As illustrated in Figure 15b, a microcavity was first aligned with a plasmonic waveguiding silver NW on a PMMA substrate through e‐beam lithography. The microcavity was then filled with QDs as the gain material to support the WGM lasing under optical excitation, with the emission strongly coupled and guided by the plasmonic mode of the tangentially aligned silver NW. Such design makes the deposition of QDs the last fabrication step and therefore minimizes possible damage during fabrication. Because of the subwavelength field confinement of the silver NW plasmonic waveguide, the output mode area of this hybrid system was only 0.008*λ* ^2^, indicating the possibility to surpass the diffraction limit and downscale the PICs. Moreover, by hybridizing silver NWs with more than one coupled microcavities, on‐chip one‐color single mode and two‐color single mode lasing systems were respectively demonstrated. QD‐based passive photonic structures were also developed with less invasive fabrication methods. For instance, a large‐area defect‐free transmission diffraction grating was successfully created by microcontact moulding of CdSe QDs. The minimum line spacing of the grating reached ≈160 nm, with the grating diffraction efficiency tunable with the QD size. Based on this grating, the in‐ and outcoupling of visible laser radiation into a single‐mode planar sol–gel waveguide was demonstrated.^[^ ^146^ ^]^

Rong et al. developed a pattern‐assisted approach to fabricate QD‐based active coherent light sources and passive optical components within one chip for functional PICs demonstration.^[^ ^143^ ^]^ As shown in Figure 15c, a precise trench pattern of the entire PIC was first defined on a PMMA film; the trench pattern was then compactly filled with core–shell CdSe/ZnS QDs. More than ten on‐chip integrated photonic components, including a waveguide‐ring resonator (WRR) laser, low‐noise amplifier, bending waveguide, Y‐splitter, Mach–Zehnder interferometer, and grating, were demonstrated within one PIC (Figure 15d). Recently, the research group used the same method to fabricate various passive nanophotonic devices containing low‐loss QD waveguides.^[^ ^144^ ^]^ For instance, a QD‐coated straight waveguide was fabricated and exhibited a transmission loss of only 36.2 dB cm^−1^ at 800 nm (the emission peak of the QD is around 650 nm), which is low enough to support a functional nanophotonic circuit with an in‐plane dimension of 65 × 60 µm^2^ (Figure 15e). The polarization and spectral range of the excitation light could be selected and modulated by the coupling gratings and the WRR structures and scattered‐out at different gratings. In addition, a transfer‐printing approach was developed to integrate a high gain red QD microplate laser on top of a green QD waveguide (Figure 15f). It was shown that the red light from the laser was guided along the waveguide with very low loss, proofing the viability of QD‐based PICs with both active and passive components.

## 6 Discussions and Prospects

We have reviewed the recent research efforts in combining photonic structures with colloidal QDs to achieve optical and electronic devices with high performances and new functions. There are a few important observations that shed light on continued research and commercialization of the technologies:

1) The integration of photonic structures and colloidal QDs not only lead to enhanced light absorption/emission, but also provides a versatile platform to manipulate properties (e.g., direction, wavelength, and polarization) of light. New functions such as emission rate modulation, nanoscale emission patterning, and on‐chip integration have been demonstrated. Low‐loss transmission has also been achieved in colloidal QD‐based passive components, indicating the promising potential of realizing all‐QD systems with on‐chip light emission, transmission, modulation, and detection.
2) Depending on different operation scenarios, the same photonic structure can be utilized to enhance either the light absorption or light emission of a device. For instance, diffraction grating nanostructures and cavity designs have been used in solar cells and LEDs, respectively, to enhance light absorption or improve light outcoupling efficiency. Similarly, metal plasmonic structures have been applied to boost the photon‐to‐electron conversion efficiency in solar cells and photodetectors, the electron‐to‐photon conversion efficiency in LEDs, and the photon‐to‐photon conversion efficiency in color converters and lasers. Such versatility suggests that the fabrication know‐hows developed for one type of devices can be readily extended to other types of devices and thus speed up the development of QD/photonic structure integrated devices as a whole.
3) While colloidal QDs are solution‐processable and can be desposited through various printing techniques, many photonic structures presented in this work are still fabricated through the labor‐intensive e‐beam lithography and dry etching methods, which increases the fabrication cost and may cause damage to QDs if applied after QD deposition. New high‐throughput and nondestructive patterning methods such as nanoimprinting, transfer‐printing, and laser manufacturing, should be explored to achieve a cost‐effective approach to integrate photonic structures with solution processed semiconductors.
4) It is noted that although some light–matter interaction theories and device simulation models were respectively developed for specific device structures, there has not been a universal model that can consistently guide the design principles of integration of photonic structures and QD‐based optoelectronic devices. Currently, the selection and optimization of QD properties and photonic structures for various device applications still require intensive experimental efforts to identify the influence of photonic structures to the optical and electronic properties of the QD active layer. More efforts should be made to develop device models that can holistically reflect the roles of optical‐tuning and electrical‐tuning in device performances. On the other hand, the rapidly developed artificial intelligence methods, such as deep learning,^[^ ^147^ ^]^ may become a powerful tool to accelerate the design of photonic structures, QD materials, and devices, together with the numerical and experimental investigations to fulfil the design principles.
5) We note that many of the integration strategies of the photonic structures presented in this review have also been applied to devices based on organic semiconductors,^[^ ^1^ ^]^ perovskite thin‐films,^[^ ^148^ ^]^ and 2D materials.^[^ ^8^ ^]^ For light‐absorbing devices such as solar cells and photodetectors, a similar range of performance enhancement was observed for different material systems, and the integration methods and enhancement principles of the photonic structures are mostly exchangeable among these systems. For the light emission enhancement and modulation, QDs show advantages in narrow emission band and strong localized excitonic properties and their radiative behaviors show sensitive response to the strong interactions between the QDs and the photonic structures. Also, applications in on‐chip photonic circuits have been explored much more extensively for QDs compared to other materials.

## Conflict of Interest

The authors declare no conflict of interest.
